# Editorial Notes

**Published:** 1938

**Authors:** 


### Editorial Notes

**Resignation of Miss E. M. Johnston, Matron, B.R.I.**

After ten years service as Matron to the Bristol Royal Infirmary, Miss E. M. Johnston has retired. At the Annual Reunion and Garden Party several presentations were made to her by the nursing staff, the laundry maids, the ward maids, the home maids, the workmen and by members of the Committee, medical staff and friends. Mrs. E. M. Harford, Chairman of the Nurse Committee, in making the presentation for the latter group, spoke of the many improvements that Miss Johnston had made in the nurses' régime—notably the system of holiday and off-duty times, whereby the nurses know six months ahead when they will be off duty and on holiday.

Miss Johnston has almost succeeded in arranging a ninety-six-hour-fortnight for nurses. She gave great support to the formation of the B.R.I. Nurses' League, obtained a hard tennis court for the Nurses' Home, established a special dietetic kitchen, and raised a fund for the restoration and redecoration of the Infirmary Chapel. At a time when there seems to be increasing difficulty in attracting recruits to the nursing profession, there is need of matrons who can contemplate innovations without dismay and can progress without losing hold of the continuity of the traditional spirit of sympathy and service which has inspired the nursing profession.

**Chair of Pathology.**

THE appointment of Professor T. B. Davie to the Chair of Pathology in the University of Liverpool has caused great regret that he should be leaving Bristol after so short a time here. Yet his stay amongst us has been long enough to allow every one who came in contact with him to appreciate his worth as a pathologist, as a colleague and a friend. He leaves with the widest expressions of good wishes for his success and happiness in his old university, which has recalled him to fill so important an office.

In his place we welcome T. F. Hewer, a Bristol graduate, who after a distinguished career in our own university held a Commonwealth Fund Fellowship at Johns Hopkins University, and was afterwards at the Kitchener School of Medicine and the Wellcome Tropical Research Station at Khartoum. He left the Sudan to work in Liverpool, where during the past year he has been acting as Professor of Pathology pending the filling of the Chair by the election of Professor Davie. Professor Hewer is assured of a warm welcome when he returns to his old medical school here.

**Association of Physicians of Great Britain and Ireland.**

THE Thirty-second Annual Meeting of the Association of Physicians of Great Britain and Ireland was held in Bristol on 3rd and 4th June. The Association visited Bristol previously in 1924, when a very successful meeting was presided over by the late Dr. George Parker, with Dr. J. A. Nixon as Secretary. At that time the University buildings had only recently been completed and were not yet available.

The present meeting was held in the Physics Theatre of the University, and the members expressed admiration of its acoustic properties, the comfort of the seating, and the convenient arrangements for exhibiting slides and other illustrations of the communications. Great interest was shown in the exhibition in the Physics Laboratory of the Bragg-Paul Pulsator, which can maintain artificial respiration for long periods, and in Professor Berry's series of brains of mental defectives. The exhibits of pathological specimens by Professor Davie, Dr. Fraser and Dr. A. L. Taylor were admirable and excellently displayed. Dr. G. B. Bush exhibited skiagrams illustrating tomography and planegraphy. Dr. Thornton demonstrated a new method of preserving solutions of adrenalin. Clinical cases were exhibited in the Old Fort House, and included some rare conditions which excited considerable discussion.

The Annual Dinner was held in the Great Hall of the University, and over 100 members and guests were present at a very successful and enjoyable function in these beautiful surroundings. The speeches were of a high standard, especially that of the Lord Mayor, whose presence was much appreciated, in replying to the toast of the City of Bristol. Great credit is due to the President, Dr. J. A. Nixon, to Dr. C. Bruce Perry (Local Secretary), and to Dr. R. C. Clarke (Dinner Secretary) for the unqualified success of the entire meeting. Thanks are due to the Vice-Chancellor for permission to hold the meeting in the University.

**History of the Royal Fort.**

THE following account of the Royal Fort was printed in the programme of the Association of Physicians:—

In 1642, when the Civil War broke out, Bristol endeavoured to keep out of the struggle. The City Council, recognizing that the City would be at the mercy of any hostile artillery on the neighbouring hills, began a semi-circular line of fortifications based on the River Avon and extending over a circumference of three miles across the northern heights. Colonel Neunes, when placed in charge of the City by the Parliament, developed this line of defence and built several forts on it. One of these on St. Michael's Hill was called the Windmill Hill Fort and afterwards the Fort Royal. When Prince Rupert in 1643 besieged Bristol none of the forts was captured, but with the capitulation of the City they were handed over, and Windmill Hill fort was extended and greatly strengthened by being converted into a pentagonal fortress, mounting 22 guns and containing magazines and barracks within its rampart and moat. Its eastern or longest side was 300 feet in length, and the rampart between 10 and 12 feet in height. Prince Rupert made it his headquarters when it was completed in 1644, and renamed it Fort Royal. There are still left an entrance gateway with a house on each side of it, one being traditionally the Governor's house: the remains of two bastions, the southern and the south-eastern, and the foundations of the rampart between them (still visible in the garden of Royal Fort House): the entrance lane from St. Michael's Hill and a fine well with a copious spring of water and a cistern holding 10,000 gallons which until recently supplied the neighbourhood.

In 1645, after the battle of Naseby, Cromwell and Fairfax invested the City. Fairfax made his headquarters at Stoke Park, Stapleton, where Stoke Park Colony is now situated. On 23rd August the garrison of Fort Royal sallied out in storms of pouring rain to attack the besiegers, but they were driven back. On 10th September Cromwell's troops made a general assault in which the eastern suburbs of Bristol were captured. At 10 a.m. after three or four hours' fierce fighting Prince Rupert requested a parley. Both parties were anxious to save the city itself from destruction, so that terms were agreed to that evening, and on the next day, 11th September, 1645, the Prince marched out of Fort Royal with colours flying, accompanied by all his troops and adherents and their baggage. Prince Rupert, mounted on a black charger and clad in scarlet and silver, was courteously accompanied by Fairfax over the Downs, and the city fell once again into the hands of Parliament. When the Civil War broke out again in 1648 Parliament voted large sums to repair the great Fort and the Castle. However, at the end of 1654, Cromwell ordered the destruction of the Castle, and in 1655 of the Great Fort. The work was costly and tedious, but at last the Fort was completely destroyed, with the exception of the gate-house, a corner tenement called "the Court of the Guard House," and a few other houses which were let as private dwellings by the Corporation in 1658-9.

Early in the eighteenth century the Tyndall family became tenants of certain houses in the Royal Fort. One of them, Thomas Tyndall, in 1753 built the beautiful mansion now known as Royal Fort House. His descendants occupied the House and the surrounding gardens until 1916, when the last surviving Miss Tyndall sold the property to Mr. H. H. Wills, who erected the present magnificent Physics Department of the University, leaving intact the Royal Fort House with its lovely gardens and the stately trees of the entrance drive.

(Compiled from the late Dr. George Parker's account of "Tyndall's Park, Bristol, Fort Royal and the Fort House therein," *Transactions of the Bristol and Gloucestershire Archæological Society,* vol. li., 1929.)

